# Randomized clinical trial with fractional CO_2_ laser and Clobetasol in the treatment of Vulvar Lichen Sclerosus: a clinic study of feasibility

**DOI:** 10.1186/s13104-023-06300-7

**Published:** 2023-03-10

**Authors:** Hakayna Calegaro Salgado, Denise Gasparetti Drumond, Gabriel Duque Pannain, Louise Gracielle de Melo e Costa, Fernanda Souza Sampaio, Isabel Cristina Gonçalves Leite

**Affiliations:** 1 Federal University of Juiz de Fora, Juiz de Fora, MG 36036-110 Brazil; 2 Department of Surgery Medical School, Federal University of Juiz de Fora, Juiz de Fora, MG 36036-110 Brazil; 3 Medical Residency, Institute of Medical Assistence to State Public Servants, São Paulo, SP 04039-000 Brazil; 4 Pathology Department, Federal University of Juiz de Fora, Juiz de Fora, MG 36036-110 Brazil; 5 Gynecology Service, University Hospital of the Federal University of Juiz de Fora, Juiz de Fora, MG 36036-110 Brazil; 6 Public Health Department, Federal University of Juiz de Fora, Juiz de Fora, MG 36036-110 Brazil

**Keywords:** Clobetasol, Vulva diseases, Vulvar Sclerosus Lichen, Laser therapy

## Abstract

**Objectives:**

The main objective of the study was to describe and compare the feasibility of using fractional CO_2_ laser to the usual treatment with Clobetasol. Randomized clinical trials brought together 20 women from a Brazilian university hospital, 9 of them were submitted to Clobetasol treatment and 11 to laser therapy. Sociodemographic data were obtained and quality of life parameters, vulvar anatomy, self-perception and histopathological analysis of vulvar biopsies were evaluated. Evaluations were made before the beginning of the treatment, during its implementation, right after its completion (3 months), and 12 months after. The SPSS 14.0 software was used, obtaining descriptive measurements. The level of significance adopted was 5%.

**Results:**

The clinical/anatomical characteristics of the vulva did not differ between the treatment groups, as much before as after its performance. There was no statistically significant difference between the treatments performed regarding the impact on the life quality of the patients. A higher satisfaction degree with the treatment was obtained with the patients in the Laser group in the third month of evaluation. Laser therapy also revealed higher occurrence of telangiectasia after treatment completion. Fractional CO2 laser has proven to be well accepted and is a promising therapeutic option.

*Registration number and name of trial registry* The institutional review board status was approved by the Research Ethics Committee of HU/ UFJF under advisory number 2881073 and registered in the Brazilian Clinical Trials, with consent under registration RBR-4p9s5y. Access link: https://ensaiosclinicos.gov.br/rg/RBR-4p9s5y

**Supplementary Information:**

The online version contains supplementary material available at 10.1186/s13104-023-06300-7.

## Introduction

Lichen Sclerosus is a rare chronic inflammatory dermatosis that affects mostly women and the etiology is still unknown [1, 2].

The most commonly affected site is the genital region [3, 4].

The lesion begins as bluish-white interfollicular papules, and then evolves into polygonal bright white papules [4]. It generally begins in the clitoral region, extending to the labia minora, vestibule, perineum, and perianal region, with significant architectural distortion of the genitalia [5].

Itching is the main symptom reported. Pain, discomfort and dysuria are also reported [6]. This is a disease that generates significant psychological disturbances in women’s lives [7].

The most characteristic alteration of the disease is the collagen homogenization zone in the dermis, also called the sclerosis zone [8].

The standard treatment consists of the use of high-potency topical corticosteroids, the most widely used being Clobetasol Propionate. However, there are still restrictions about the long-term use of the medication [9].

This study has as main purpose to compare the results of the treatment of Lichen Sclerosus with Clobetasol and fractional CO_2_ laser.

## Method

This is a randomized clinical trial to assess the feasibility and equivalence of fractional CO_2_ laser compared to standard treatment with Clobetasol, approved by the Research Ethics Committee of HU/ UFJF (Portuguese acronyms) (advisory number 2881073) and registered in the Brazilian Clinical Trials (RBR-4p9s5y).

The sample consisted of 20 women assisted at the vulva clinic of the Gynecology Service of HU/ UFJF.

For the sample calculation of an experimental study with an estimate of the proportion for 2 independent groups, with the purpose of evaluating the equivalence of the laser and the standard treatment, the following parameters were accepted: alpha error of 5%, test power 95%, success estimation in the control group of 40% and 1:1 ratio. A minimum sample size of 20 subjects (10 in each group) was estimated. Women with Vulvar Lichen Sclerosus diagnosed by histopathological evaluation of vulvar biopsy were included, and women who were already under treatment for this condition at the time of the survey or those who had used any form of treatment for the disease in 30 days prior to the outpatient appointment were excluded from the survey. Women with other diagnoses of vulvar disease identified in the biopsy were also excluded.

Additional file 1: Fig. S1 describes the recruitment and follow-up of the patients.

The study was developed in the period between March 27, 2019 and March 18, 2020, with completion of the proposed treatment. The patients were reevaluated 12 months after the end of the treatment.

After signing the Informed Consent Form, the identification form was filled out, obtaining epidemiological data. Then the patients answered the WHOQOL-BREF questionnaire [10] and were then referred to treatment, being divided into two groups: Laser group and Clobetasol group. Two researchers performed the randomization randomly drawing the patients between the groups.

The patients in the Clobetasol group were instructed to use a daily night application of Clobetasol ointment (Clobetasol Propionate 0.05%) in the first month, followed by night applications on alternate days in the second month, and a night application on two consecutive days of the same week (Saturdays and Sundays) in the third month. Since then, the use was guided according to the patient's needs, not exceeding twice a week.

Patients in the Laser group underwent three sessions of fractional CO_2_ laser (SmartXide2 system, Monalisa Touch, DEKA Laser, Florence, Italy), with a 30-day interval between each session [11]. The patients received a local anesthetic ointment (7% lidocaine and 7% tetracaine) before the laser application. The sessions were performed in the following settings: power 25 watts/Stack 1/ime 700 microseconds/Spacing 700 micromillimeters. The application was held by another member of the research group.

The groups were followed up on a monthly basis. During each follow-up visit, the patient was examined by the responsible researcher, not knowing the patient's treatment group, with completion of the vulva morphological/anatomical manifestations evaluation form, according to the criteria proposed by Sheinis and Selk [12], and obtaining photographs of the vulvar region.

After the end of the treatment, all patients underwent a new vulva biopsy, in a contralateral region to the first biopsy performed and answered the life quality questionnaire again, in addition to a questionnaire developed by the researchers themselves to evaluate the improvement of symptoms, satisfaction with the treatment, and the degree of difficulty found to perform it. These forms of evaluation were defined and chosen due to the absence of validated dimensions in the country with the same objective.

Subsequently, a new evaluation was performed 12 months after the end of the instituted treatment; with the application of quality of life questionnaires, and the patient's perception of the treatment.

The data were entered into the SPSS 14.0 software. Categorical variables were described as absolute and relative frequencies.

Participants who lost follow-up appointments were considered as flaws and therefore their statuses were kept similar to the last assessment for all variables.

For the association between categorical independent variables (sociodemographic, clinical, and patient perception) and dependent variable (type of treatment) Pearson's chi-square test was used, complemented with Fisher's test, when necessary. In the case of quantitative variables, normality was evaluated using the Kolmogorov–Smirnov test, and normality was assumed. Thus, quantitative variables (current and diagnosed ages and domains of the WHOQOL-BREF) were associated with the type of treatment by the Student's t-test.

Spearman correlation was performed to assess the relationship between the extent of vulvar disease and the patient's perception before and after treatment.

The Wilcoxon test for dependent samples and ordinal responses was used to assess histopathological characteristics before and after treatment. The significance level was 5%.

## Results

The sample was made up of elderly women (100%), mostly white (60%), married (55%), and with up to 9 years of schooling (60%).

The most affected site by Vulvar Lichen Sclerosus was the lower third of the right labia majora (25% of cases).

The clinical/anatomical characteristics of the vulva did not differ between the groups, both before and after treatment (p > 0.05), except for erosion, which was more frequent before treatment in the Laser group (p < 0.04), and mild forms of hyperkeratosis and lichenification, which were also more frequent in the Laser group after three months of treatment (p < 0.03) (Additional file 3: Table S1 shows the clinical characteristics of the patients according to the time and type of treatment instituted).

There were no statistically significant differences between the treatment groups in the assessment of the WHOQOL-BREF domains, with the exception of the Social domain in favor of treatment with Clobetasol (p < 0.05) (Table 1).

**Table 1:** Mean and Standard Deviation according to the domains of the WHOQOL-BREF questionnaire in patients with Vulvar Lichen Sclerosus undergoing treatment

Variable	Physical µ (SD)	Psychological µ (SD)	Social µ (SD)	Environmental µ (SD)
First evaluation treatment				
Laser	3.80 (0.72)	3.51 (0.75)	3.12 (0.95)	3.52 (0.33)
Clobetasol	3.49 (0.96)	3.61 (0.55)	3.26 (0.88)	3.33 (0.43)
p-value	0.342	0.408	0.762	0.542
Second evaluation (after 3 months of treatment)				
Treatment				
Laser	3.90 (0.65)	3.45 (0.77)	2.88 (0.40)	3.55 (0.30)
Clobetasol	3.49 (0.63)	3.57 (0.64)	2.92 (0.70)	3.42 (0.54)
p-value	0.587	0.373	0.049	0.252

Source: The authors (2020)

*µ* Mean, *SD* Standard Deviation

The extent of vulvar disease before treatment showed a negative impact on quality of life, with lower scores in the Environment domain of the WHOQOL-BREF questionnaire (p = 0.042) (Additional file 4: Table S2 shows the relationship between vulvar involvement before treatment and the score obtained in the Environment domain).

Only 35% of patients reported having an active sexual life. After 3 months of the proposed treatment, women without a sexual partner (p < 0.01) and with higher education (p = 0.05) obtained better evaluation in the Psychological domain and, on the other hand, women with a sexual partner (p < 0.01) better performance in the Social domain of the WHOQOL-BREF.

The presence of vulvar ulceration (p = 0.052) and hyperkeratosis (p = 0.021) before treatment negatively influenced the self-perception of vulvar appearance after 3 months of treatment. Perianal involvement (p = 0.031) and the presence of synechiae (p = 0.048) negatively influenced the quality of sexual activity evaluated also after the end of treatment. The narrowing of the vaginal introitus, in turn, had a negative influence on the self-perception of Itching (p = 0.044) after 3 months of treatment (Additional file 5: Table S3 shows the relationship between the presence of clinical signs of the disease and the patient's self-perception about the treatment).

There was a higher degree of satisfaction in the group treated with Laser in terms of patient self-perception (p = 0.006). However, this difference was not maintained at the 12-month evaluation (Table 2).

**Table 2:** Mean and p-value according to the perception of patients with Vulvar Lichen Sclerosus regarding the aspects of the treatment to which they were submitted

Variable	Averages per items of evaluation µ (SD)						
	Itching	Dysuria	Pain/sting	Sexual Activity	Appearance	Satisfaction	Difficulty
Evaluation after 3 months of treatment							
Laser	9.18 (0.87)	8.80 (2.90)	9.18 (0.75)	6.67 (2.89)	8.82 (2.40)	9.82 (0.40)	2.64 (0.83)
Clobetasol	8.44 (1.88)	9.33 (1.66)	9.00 (1.66)	6.50 (3.70)	8.50 (0.93)	9.33 (1.32)	2.56 (1.03)
p-value	0.204	0.283	0.187	0.682	0.242	0.006	0.615

Source: The authors (2020)

*µ* Mean, *SD* Standard Deviation

The evaluation 12 months after treatment revealed a higher occurrence of mild cases of hyperkeratosis and lichenification (p = 0.02), in addition to excoriation (p < 0.03) in the Laser group. The situation of the WHOQOL-BREF domains also did not differ between the evaluated groups. Regarding the degree of patient satisfaction, the highest degree of satisfaction obtained in the Laser group was not maintained after 12 months of treatment.

Additional file 2: Fig. S2 shows the results after instituting treatment with three sessions of fractionated CO2 laser.

## Discussion

The great challenge for physicians lies in the management of patients with Lichen Sclerosus who do not respond to topical treatment.

Symptoms often reappear after discontinuation of topical therapy, and a maintenance regimen is indicated [13].

Although the anatomical and morphological characteristics of the vulva assessed in the study did not undergo major changes or regression after both treatments, the fractional CO_2_ laser showed significant short-term improvement in the treatment of hyperkeratosis and atrophy.

Reduction of hyperkeratosis can be justified by the fact that laser therapy promotes the ablation of the dermo-epidermal zone with inadequate function, creating a new region with correct function, allowing the remission of the disease [14].

A recently published study conducted in Italy evaluated 40 women with Lichen Sclerosus refractory, regarding long-acting topical high-potency corticosteroid treatment who underwent 2 sessions of CO_2_ laser in the vulvar region, associated with intravaginal laser therapy in those postmenopausal women with vaginal symptoms. Considerable improvement was noted in vulvar pruritus, vulvar dryness, superficial dyspareunia, and improved sensitivity during intercourse, with the improvement being progressive after the second session [15].

A retrospective study was also conducted, to review the short-term effects and safety of fractional CO_2_ laser therapy in patients with symptoms of vulvovaginal atrophy through questionnaires and visual analogue scale. 139 patients were evaluated, who received three laser sessions 6 weeks apart and were followed up for an average period of 13.8 weeks. Among the patients evaluated, 22% were diagnosed with Lichen Sclerosus. The study observed considerable improvement in all means of assessment after the treatment was carried out [16].

It is believed that the higher degree of satisfaction with the fractional CO_2_ laser treatment is due to the convenience of performing the treatment, not requiring daily applications of medication and manipulation of the vulva.

Vulvar Lichen Sclerosus is a disease that is difficult to treat and often requires long-term maintenance therapy. In our evaluation after 12 months of treatment, we observed that there were no statistically significant differences in terms of quality of life and in most clinical/anatomical alterations of the vulva between the compared groups. The higher degree of satisfaction with the treatment in the Laser group observed soon after the end of the treatment was also not maintained in the one-year evaluation. This may be due to the effects of laser therapy that fade over time, and a new rescue session may be required. However, the available literature still does not present well-defined protocols on the number and intervals between sessions intended for the treatment of Lichen Sclerosus Vulvar.

Lichen Sclerosus also negatively affects the patient's quality of life. From all fields of life, sexual function is shown to be the most negatively affected by the disease [5]. In our study, in the evaluation of patient self-perception, sexual intercourse was the criterion with the lowest score.

The association of intravaginal laser with vulvar treatment is an important point that could change some of the results of our study, considering the age range of involvement of Lichen Sclerosus, with important symptoms resulting from hypoestrogenism.

The greater the occurrence of vulvar signs of the disease, the worse the patient's perception of the appearance of her genitalia. A recent study demonstrated that women with Lichen Sclerosus scored significantly lower on a genital self-image scale [17].

Women without a sexual partner and with higher education obtained higher scores in the Psychological domain. A study showed that patients evaluated for sexual difficulties reported that many of the obstacles are related to behaviors and attitudes of their partners and the presence of disease [18].

In Social domain, patients with a sexual partner scored better, probably due to the support network that the family formed with their partner generates for the patient [10].

The treatment of Lichen Sclerosus has not yet evolved beyond topical corticosteroids. However, this study, as well as the others already conducted, shows positive and promising results in the use of fractional CO_2_ laser [19, 20].

Fractional CO_2_ laser is a promising therapeutic option especially in patients who barely or partially respond to treatment with Clobetasol. It has been shown to be well accepted by patients. However, the duration of its effect is still a question to be defined, as well as the number of sessions to be performed and the possibility of adopting a maintenance regimen.

### Limitation

The major limitation of the study is in the sample size. The difficulty in the size of the sample, besides the fact that this is a rare disease, lies in the need for a long term follow-up.

## Supplementary Information

**Additional file 1: Figure S1.** Monitoring Flowchart.

**Additional file 2: Figure S2.** Comparison before and after the institution of treatment with three sessions of fractionated CO_2_ laser for Vulvar Lichen Sclerosus.

**Additional file 3: Table S1.** Clinical characteristics of patients with Vulvar Lichen Sclerosus, according to treatment. March 2019 to 2020.

**Additional file 4: Table S2.** Relationship between vulvar involvement before treatment and the score obtained in the Environment domain of the WHOQOL-BREF 3 months after treatment.

**Additional file 5: Table S3.** Relationship between the presence of clinical signs of Lichen Sclerosus Vulvar and the patient's self-perception about the treatment.

## Data Availability

The datasets used and/or analyzed during the current study are available from the corresponding author on reasonable request.
