# Supplementary material for: Randomized clinical trial with fractional CO2 laser and Clobetasol in the treatment of Vulvar Lichen Sclerosus: a clinic study of feasibility
Source: BMC Res Notes. 2023 Mar 10;16:33. doi: 10.1186/s13104-023-06300-7 (PMC9999649; doi:10.1186/s13104-023-06300-7)
Supplement: Supplementary file 1 — Additional file 1: Figure S1. Monitoring Flowchart. [file 13104_2023_6300_MOESM1_ESM.docx]

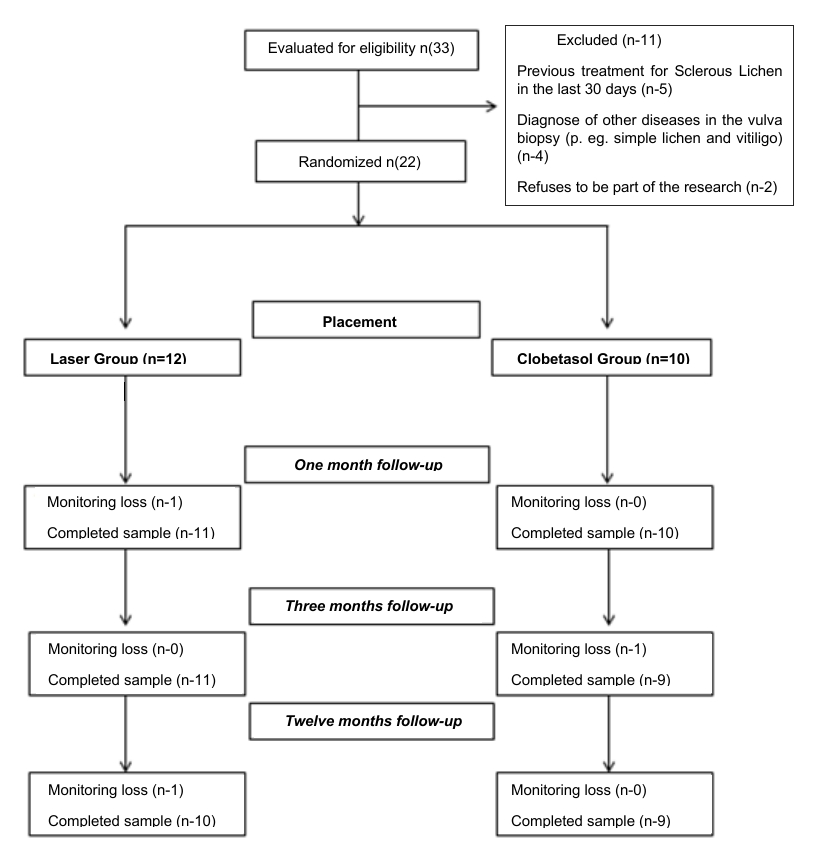
Figure 1 – Monitoring Flowchart

Reasons for patients’ loss: **Laser Group** - occurrence of acute pulmonary edema, with discontinuation of treatment in one of the patients (after 1 month of follow-up) and impossibility of telephone contact with the number provided in medical records by another patient (after 12 months of follow-up). / **Clobetasol Group** – quit participating in the research, without any specific reason, wishing only to maintain the proposed treatment.

Source: The authors (2020).
