# Supplementary material for: Randomized clinical trial with fractional CO2 laser and Clobetasol in the treatment of Vulvar Lichen Sclerosus: a clinic study of feasibility
Source: BMC Res Notes. 2023 Mar 10;16:33. doi: 10.1186/s13104-023-06300-7 (PMC9999649; doi:10.1186/s13104-023-06300-7)
Supplement: Supplementary file 2 — Additional file 2: Figure S2. Comparison before and after the institution of treatment with three sessions of fractionated CO2 laser for Vulvar Lichen Sclerosus. [file 13104_2023_6300_MOESM2_ESM.docx]

Figure 2 - Comparison before and after the institution of treatment with three sessions of fractionated CO_2_ laser for Vulvar Lichen Sclerosus

**A)**
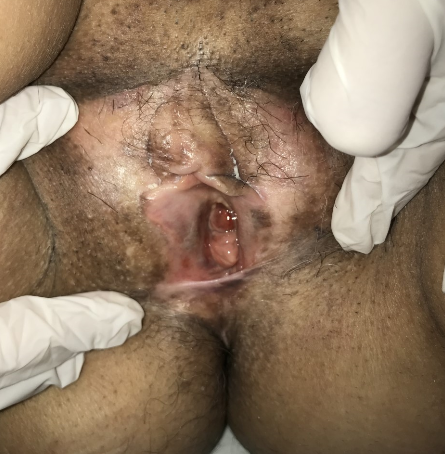
**B)**
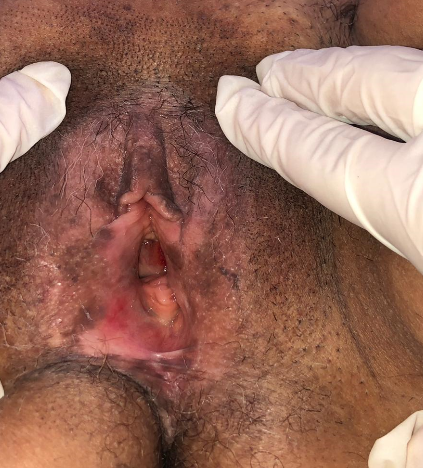


Legend: **A)** Patient with Vulvar Lichen Sclerosus before the institution of treatment. **B)** Aspect of the vulva after 3 sessions of Fractional CO2 Laser, being identified improvement of hypochromia, skin atrophy and hyperkeratosis areas.

Source: Author's personal file (2020).
