# Supplementary material for: Randomized clinical trial with fractional CO2 laser and Clobetasol in the treatment of Vulvar Lichen Sclerosus: a clinic study of feasibility
Source: BMC Res Notes. 2023 Mar 10;16:33. doi: 10.1186/s13104-023-06300-7 (PMC9999649; doi:10.1186/s13104-023-06300-7)
Supplement: Supplementary file 3 — Additional file 3: Table S1. Clinical characteristics of patients with Vulvar Lichen Sclerosus, according to treatment. March 2019 to 2020. [file 13104_2023_6300_MOESM3_ESM.docx]

**Table 1 -** Clinical characteristics of patients with Vulvar Lichen Sclerosus, according to treatment. March 2019 to 2020.

| **Characteristics** | | **CO_2_ fractional laser** | | | | | | **Clobetasol** | | **CO_2_ fractional laser** | | | | | | **Clobetasol** | | | | | | | | |  |
| --- | --- | --- | --- | --- | --- | --- | --- | --- | --- | --- | --- | --- | --- | --- | --- | --- | --- | --- | --- | --- | --- | --- | --- | --- | --- |
| **Extension of the disease** | | **First Evaluation**  **(before treatment)**  **n (%) n(%)** | | | | | | | | | **Fourth Evaluation (after three months of treatment)**  **n (%) n (%)** | | | | | | | | | | | | |  |  |
|  |  |  | | | | | | | | |  |  |  |  |  |  |  |  |  |  |  |  |  |  |  |
| “Among eight” | | | 6 (54.5) | | | | | 2 (22.2) | | | 6 (54.5) | | | | | | | | | | | 2 (22.2)  5 (55.6)  2 (22.2) | |  |  |
| Labia e clitoris  Labia, clitoris e perinea | | | 2 (18.2)  3 (27.3) | | | | | 5 (55.6)  2 (22.2) | | | 2 (18.2)  3 (27.3) | | | | | | | | | | |  |  |  |  |
| **p-value** | | | 0.190 | | | | | | | | 0.190 | | | | | | | | | | | | |  |  |
| **Fissure** | |  | | | | | |  | | |  | | | | | | | | | | |  | |  |  |
| Light | | 3 (27.3) | | | | | | 2 (22.2) | | | 2 (18.2) | | | | | | | | | | | 0 (0.0) | |  |  |
| Moderate  Severe  Absent | | 2 (18.2)  1 (9.0)  5 (45.5) | | | | | | 0 (0.0)  0 (0.0)  7(77.8) | | | 0 (0.0)  0 (0.0)  9 (81.8) | | | | | | | | | | | 0 (0.0)  0 (0.0)  9 (100.0) | |  |  |
| **p-value** | | 0.338 | | | | | | | | | 0.479 | | | | | | | | | | | | |  |  |
| **Hypochromic** | |  | | | | | |  | | |  | | | | | | | | | | |  | |  |  |
| Light | | 2 (18.1) | | | | | | 5 (55.6) | | | 4 (36.4) | | | | | | | | | | | 4 (44.5) | |  |  |
| Moderate  Severe  Absent | | 4 (36.4)  5 (45.5)  0 (0.0) | | | | | | 2 (22.2)  2 (22.2)  0 (0.0) | | | 5 (45.5)  2 (18.1)  0 (0.0) | | | | | | | | | | | 1 (11.1)  2 (22.2)  2 (22.2) | |  |  |
| **p-value** | | 0.216 | | | | | | | | | 0.211 | | | | | | | | | | | | |  |  |
| **Skin Thinning** | |  | | | | | |  | | | | |  | | | |  | | | | | | |  |  |
| Light | 0 (0.0) | | | | | 1 (11.1) | | | | | | | 4 (36.4) | | | | | | | | 5 (55.6) | | |  |  |
| Moderate  Severe  Absent | 6 (54.5)  5 (45.5)  0 (0.0) | | | | | 6 (66.7)  2 (22.2)  0 (0.0) | | | | | | | 5 (45.5)  2 (18.1)  0 (0.0) | | | | | | | | 3 (33.3)  0 (0.0)  1 (11.1) | | |  |  |
| **p-value** | 0.349 | | | | | | | | | |  | | | | | | | | | | | 0.328 | |  |  |
|  | |  | | | | | | | | |  | | | | | | | | | | |  | |  |  |
| **Erosion** | |  | | | | | | | | |  | | | | | | | | | | |  | |  |  |
| Light | 5 (45.5) 0 (0.0) | | | | | | | | | | | | | 1 (9.1) | | | | 0 (0.0) | | | | | |  |  |
| \| Moderate \| \| 0 (0.0) \| \| \| \| \| \| \| 0 (0.0) \| \| \| \| \| \| \| \| \| \| \| \| \| \| \| \| \| \| \| \| \| 0 (0.0) \| \| \| \| \| \| \| \| \| \| \| \| 0 (0.0) \| \| \| \| \| \| \| --- \| --- \| --- \| --- \| --- \| --- \| --- \| --- \| --- \| --- \| --- \| --- \| --- \| --- \| --- \| --- \| --- \| --- \| --- \| --- \| --- \| --- \| --- \| --- \| --- \| --- \| --- \| --- \| --- \| --- \| --- \| --- \| --- \| --- \| --- \| --- \| --- \| --- \| --- \| --- \| --- \| --- \| --- \| --- \| --- \| --- \| --- \| --- \| \| Severe \| \| 0 (0.0) \| \| \| \| \| \| \| 0 (0.0) \| \| \| \| \| \| \| \| \| \| \| \| \| \| \| \| \| \| \| \| \| 0 (0.0) \| \| \| \| \| \| \| \| \| \| \| \| 0 (0.0) \| \| \| \| \| \| \| Absent \| \| 6(54.5) \| \| \| \| \| \| \| 9 (100.0) \| \| \| \| \| \| \| \| \| \| \| \| \| \| \| \| \| \| \| \| \| 10(90.9) \| \| \| \| \| \| \| \| \| \| \| \| \| \| \| 9(100.0) \| \| \| \| **p-value** \| \| \| \| \| \| \| \| \| **0.038** \| \| \| \| \| \| \| \| \| \|  \| \| \| \| \| \| \| \| \| \| \| \| \| 0.353 \| \| \| \| \| \| \| \| \| \| \| \| \| \|  \| \| \|  \| \| \| \| \| \| \| \| \|  \| \| \| \| \| \| \| \| \| \|  \| \| \| \| \| \| \| \| \| \| \| \| \|  \| \| \|  \| \| \| \| \| \| \| \| \| \| \| \| \| \| **Ulceration** \| \| \| \| \| \| \| \| \|  \| \| \| \| \| \| \| \| \| \|  \| \| \| \| \| \| \| \| \| \| \| \| \|  \| \| \|  \| \| \| \| \| \| \| \| \| \| \| \| \| \| Light \| \| 3 (27.3) \| \| \| \| \| \| \| \| \| 0 (0.0) \| \| \| \| \| \| \| \| \| \| \| \| \| \| \| \| \| \| \| 1(9.1) \| \| \| \| \| \| \| 1(11.1) \| \| \| \| \| \| \| \| \| \| \| \| Moderate \| \| 0 (0.0) \| \| \| \| \| \| \| \| \| 0 (0.0) \| \| \| \| \| \| \| \| \| \| \| \| \| \| \| \| \| \| \| 0 (0.0) \| \| \| \| \| \| \| 0 (0.0) \| \| \| \| \| \| \| \| \| \| \| \| Severe \| \| 0 (0.0) \| \| \| \| \| \| \| \| \| 0 (0.0) \| \| \| \| \| \| \| \| \| \| \| \| \| \| \| \| \| \| \| 0 (0.0) \| \| \| \| \| \| \| 0 (0.0) \| \| \| \| \| \| \| \| \| \| \| \| Absent \| \| 8(72.7) \| \| \| \| \| \| \| \| \| 9(100.0) \| \| \| \| \| \| \| \| \| \| \| \| \| \| \| \| \| \| \| 10(90.9) \| \| \| \| \| \| \| 8(88.9) \| \| \| \| \| \| \| \| \| \| \| \| **p-value** \| \| \| \| \| \| \| \| \| \| \| \| 0.218 \| \| \| \| \| \|  \| \| \| \| \| \| \| \| \| \|  \| \| \| 0.881 \| \| \| \| \| \| \| \| \| \| \| \| \| \| \| \| \| \|  \| \| \| \| \| \| \| \| \| \| \| \|  \| \| \| \| \| \|  \| \| \| \| \| \| \| \| \| \|  \| \| \|  \| \| \| \| \| \| \| \| \| \| \| \| \| \| \| \| \| \| **Hyperkeratosis** \| \| \| \| \| \| \| \| \| \| \| \|  \| \| \| \| \| \|  \| \| \| \| \| \| \| \| \| \|  \| \| \|  \| \| \| \| \| \| \| \| \| \| \| \| \| \| \| \| \| \| Light \| \| 6 (54.5) \| \| \| \| \| \| \| \| \| \| \| \| 5(55.6) \| \| \| \| \| \| \| \| \| \| \| \| \| \| \| \| \| 9(81.8) \| \| \| \| \| 3(33.3) \| \| \| \| \| \| \| \| \| \| \| \| \| Moderate \| \| 3(27.3) \| \| \| \| \| \| \| \| \| \| \| \| 4(44.4) \| \| \| \| \| \| \| \| \| \| \| \| \| \| \| \| \| 1(9.1) \| \| \| \| \| 0(0.0) \| \| \| \| \| \| \| \| \| \| \| \| \| Severe \| \| 2(18.2) \| \| \| \| \| \| \| \| \| \| \| \| 0(0.0) \| \| \| \| \| \| \| \| \| \| \| \| \| \| \| \| \| 0(0.0) \| \| \| \| \| 0(0.0) \| \| \| \| \| \| \| \| \| \| \| \| \| Absent \| \| 0(0.0) \| \| \| \| \| \| \| \| \| \| \| \| 0(0.0) \| \| \| \| \| \| \| \| \| \| \| \| \| \| \| \| \| 1(9.1) \| \| \| \| \| 6(66.7) \| \| \| \| \| \| \| \| \| \| \| \| \| **p-value** \| \| \| \| \| \| \| 0.358 \| \| \| \| \| \| \| \| \| \| \| \| \| \| \| \|  \| \| \| \| \|  \| \| \| **0.024** \| \| \| \| \| \| \| \| \| \| \| \| \| \| \| \| \| \|  \| \| \| \| \| \| \|  \| \| \| \| \| \| \| \| \| \| \| \| \| \| \| \|  \| \| \| \| \|  \| \| \|  \| \| \| \| \| \| \| \| \| \| \| \| \| \| \| \| \| \| **Excoriation** \| \| \| \| \| \| \| \| \| \| \| \| \| \| \| \| \| \| \| \| \| \| \| \| \| \| \| \| \| \| \| \| \| \| \| \| \| \| \| \| \| \| \| \| \| \| \| \| \| Light 7(63.6) 5(55.6) 5(45.5) 0(0.0) \| \| \| \| \| \| \| \| \| \| \| \| \| \| \| \| \| \| \| \| \| \| \| \| \| \| \| \| \| \| \| \| \| \| \| \| \| \| \| \| \| \| \| \| \| \| \| \| \| Moderate \| \| 1(9.1) \| \| \| \| \| \| \| \| \| \| \| \| \| \| \| \| \| \| 0(0.0) \| \| \| \| \| \| \| 0(0.0) \| \| \| \| \| \| \| \| \| \| \| \| 0(0.0) \| \| \| \| \| \| \| \| \| \| Severe \| \| 3(27.3) \| \| \| \| \| \| \| \| \| \| \| \| \| \| \| \| \| \| 4(44.4) \| \| \| \| \| \| \| 0(0.0) \| \| \| \| \| \| \| \| \| \| \| \| 0(0.0) \| \| \| \| \| \| \| \| \| \| Absent \| \| 0(0.0) \| \| \| \| \| \| \| \| \| \| \| \| \| \| \| \| \| \| 0(0.0) \| \| \| \| \| \| \| 0(0.0) \| \| \| \| \| \| \| \| \| \| \| \| 9(100.0) \| \| \| \| \| \| \| \| \| \| **p-value** \| \| \| \| 0.525 \| \| \| \| \| \| \| \| \| \| \| \| \|  \| \| \| \| \| \| \| \| \| \| \| \| **0.038** \| \| \| \| \| \| \| \| \| \| \| \| \| \| \| \|  \| \| \| \|  \| \| \| \|  \| \| \| \| \| \| \| \| \| \| \| \| \|  \| \| \| \| \| \| \| \| \| \| \| \|  \| \| \| \| \| \| \| \| \| \| \| \| \| \| \| \|  \| \| \| \| **Lichenification** \| \| \| \|  \| \| \| \| \| \| \| \| \| \| \| \| \|  \| \| \| \| \| \| \| \| \| \| \| \|  \| \| \| \| \| \| \| \| \| \| \| \| \| \| \| \|  \| \| \| \| Light \| 8 (72.7) \| \| \| \| \| \| \| \| \| \| \| \| \| \| \| \| \| \| \| \| \| 6 (66.7) \| \| \| \| 8 (72.7) \| \| \| \| \| \| \| \| \| \| \| \| \| \| \| \| \| 2 (22.2) \| \| \| \| \| Moderate \| 2 (18.2) \| \| \| \| \| \| \| \| \| \| \| \| \| \| \| \| \| \| \| \| \| 2 (22.2) \| \| \| \| 1 (9.1) \| \| \| \| \| \| \| \| \| \| \| \| \| \| \| \| \| 0 (0.0) \| \| \| \| \| Severe \| 1 (9.1) \| \| \| \| \| \| \| \| \| \| \| \| \| \| \| \| \| \| \| \| \| 0 (0.0) \| \| \| \| 0 (0.0) \| \| \| \| \| \| \| \| \| \| \| \| \| \| \| \| \| 0 (0.0) \| \| \| \| \| Absent \| 0 (0.0) \| \| \| \| \| \| \| \| \| \| \| \| \| \| \| \| \| \| \| \| \| 1 (11.1) \| \| \| \| 2 (18.2) \| \| \| \| \| \| \| \| \| \| \| \| \| \| \| \| \| 7 (77.8) \| \| \| \| \| **p-value** \| \| \| \| 0.551 \| \| \| \| \| \| \| \| \| \| \| \| \|  \| \| \| \| \| \| \| \| \| \| \| \|  \| \| \| \| **0.027** \| \| \| \| \| \| \| \| \| \| \| \| \| \| \| \|  \| \| \| \|  \| \| \| \| \| \| \| \| \| \| \| \| \|  \| \| \| \| \| \| \| \| \| \| \| \|  \| \| \| \| \| \| \| \| \| \| \| \| \| \| \| \|  \| \| \| \| **Elasticity loss** \| \| \| \|  \| \| \| \| \| \| \| \| \| \| \| \| \|  \| \| \| \| \| \| \| \| \| \| \| \|  \| \| \| \| \| \| \| \| \| \| \| \| \| \| \| \|  \| \| \| \| Light \| \| 0(0.0) \| \| \| \| \| \| \| \| \| \| \| \| \| \| \| \| 1(11.1) \| \| \| \| \| \| \| 1(9.1) \| \| \| \| \| \| \| \| \| \| \| \| \| \| \| \| \| \| \| 2(22.2) \| \| \| \| \| Moderate \| \| 3(27.3) \| \| \| \| \| \| \| \| \| \| \| \| \| \| \| \| 3(33.3) \| \| \| \| \| \| \| 6(54.5) \| \| \| \| \| \| \| \| \| \| \| \| \| \| \| \| \| \| \| 4(44.5) \| \| \| \| \| Severe \| \| 8(72.7) \| \| \| \| \| \| \| \| \| \| \| \| \| \| \| \| 5(55.6) \| \| \| \| \| \| \| 4(6.4) \| \| \| \| \| \| \| \| \| \| \| \| \| \| \| \| \| \| \| 2(22.2) \| \| \| \| \| Absent \| \| 0(0.0) \| \| \| \| \| \| \| \| \| \| \| \| \| \| \| \| 0(0.0) \| \| \| \| \| \| \| 0(0.0) \| \| \| \| \| \| \| \| \| \| \| \| \| \| \| \| \| \| \| 1(11.1) \| \| \| \| \| **p-value** \| \| \| \| 0.471 \| \| \| \| \| \| \| \| \| \| \| \| \|  \| \| \| \| \| \| \| \| \| \| \| \| 0.528 \| \| \| \| \| \| \| \| \| \| \| \| \| \| \| \|  \| \| \| \| **Sclerosis** \|  \| \| \| \| \| \| \| \| \| \| \| \| \|  \| \| \| \| \| \| \| \| \| \| \| \| \|  \| \| \| \| \| \| \| \| \| \| \| \|  \| \| \| \| \| \| \| \| \| \| Light \| 1 (9.1) \| \| \| \| \| \| \| \| \| \| \| \| 5 (55.6) \| \| \| \| \| \| \| \| \| \| \| \| \| \| 3 (27.3) \| \| \| \| \| \| \| \| \| \| \| \| \| \| \| \| \| 4 (44.4) \| \| \| \| \| Moderate \| 9(81.8) \| \| \| \| \| \| \| \| \| \| \| \| 4(44.4) \| \| \| \| \| \| \| \| \| \| \| \| \| \| 7(63.6) \| \| \| \| \| \| \| \| \| \| \| \| \| \| \| \| \| 4 (44.4) \| \| \| \| \| Severe \| 1 (9.1) \| \| \| \| \| \| \| \| \| \| \| \| 0 (0.0) \| \| \| \| \| \| \| \| \| \| \| \| \| \| 1 (9.1) \| \| \| \| \| \| \| \| \| \| \| \| \| \| \| \| \| 0 (0.0) \| \| \| \| \| Absent \| 0 (0.0) \| \| \| \| \| \| \| \| \| \| \| \| 0(0.0) \| \| \| \| \| \| \| \| \| \| \| \| \| \| 0 (0.0) \| \| \| \| \| \| \| \| \| \| \| \| \| \| \| \| \| 1 (11.2) \| \| \| \| \| **p-value** \| 0.066 \| \| \| \| \| \| \| \| \| \| \| \| \| \| \| \| \| \| \| \| \| \| \| \| \| \| 0.425 \| \| \| \| \| \| \| \| \| \| \| \| \| \| \| \| \| \| \| \| \| \|  \|  \| \| \| \| \| \| \| \| \| \| \| \| \| \| \| \| \| \| \| \| \| \| \| \| \| \|  \| \| \| \| \| \| \| \| \| \| \| \| \| \| \| \| \| \| \| \| \| \| **Telangiectasia** \|  \| \| \| \| \| \| \| \| \| \| \| \| \| \| \| \| \| \| \| \| \| \| \| \| \| \|  \| \| \| \| \| \| \| \| \| \| \| \| \| \| \| \| \| \| \| \| \| \| Light \| 0(0.0) \| \| \| \| \| \| \| \| \| \| \| \| \| \| \| \| \| \| \| 1(11.1) \| \| \| \| \| \| \| 1(9.1) \| \| \| \| \| \| \| \| \| \| \| \| \| 0(0.0) \| \| \| \| \| \| \| \| \| Moderate \| 0(0.0) \| \| \| \| \| \| \| \| \| \| \| \| \| \| \| \| \| \| \| 0(0.0) \| \| \| \| \| \| \| 0(0.0) \| \| \| \| \| \| \| \| \| \| \| \| \| 0(0.0) \| \| \| \| \| \| \| \| \| Severe \| 0(0.0) \| \| \| \| \| \| \| \| \| \| \| \| \| \| \| \| \| \| \| 0(0.0) \| \| \| \| \| \| \| 0(0.0) \| \| \| \| \| \| \| \| \| \| \| \| \| 0(0.0) \| \| \| \| \| \| \| \| \| Absent \| 11(100.0) \| \| \| \| \| \| \| \| \| \| \| \| \| \| \| \| \| \| \| 8(88.9) \| \| \| \| \| \| \| 10(90.9) \| \| \| \| \| \| \| \| \| \| \| \| \| 9(100.0) \| \| \| \| \| \| \| \| \| **p-value** \| 0.450 \| \| \| \| \| \| \| \| \| \| \| \| \| \| \| \| \| \| \| \| \| \| \| \| \| \| 0.353 \| \| \| \| \| \| \| \| \| \| \| \| \| \| \| \| \| \| \| \| \| \|  \|  \| \| \| \| \| \| \| \| \| \| \| \| \| \| \| \| \| \| \| \| \| \| \| \| \| \|  \| \| \| \| \| \| \| \| \| \| \| \| \| \| \| \| \| \| \| \| \| \| **Clitoris burial** \|  \| \| \| \| \| \| \| \| \| \| \| \| \| \| \| \| \| \| \| \| \| \| \| \| \| \|  \| \| \| \| \| \| \| \| \| \| \| \| \| \|  \| \| \| \| \| \| \| \| Light \| 2(18.1) \| \| \| \| \| \| \| \| \| \| \| \| \| \| \| \| \| \| \| \| 3(33.3) \| \| \| \| \| \| 2(18.1) \| \| \| \| \| \| \| \| \| \| \| \| \| \| 3(33.3) \| \| \| \| \| \| \| \| Moderate \| 4(36.4) \| \| \| \| \| \| \| \| \| \| \| \| \| \| \| \| \| \| \| \| 2(22.2) \| \| \| \| \| \| 5(45.5) \| \| \| \| \| \| \| \| \| \| \| \| \| \| 2(22.2) \| \| \| \| \| \| \| \| Severe \| 5(45.4) \| \| \| \| \| \| \| \| \| \| \| \| \| \| \| \| \| \| \| \| 4(44.5) \| \| \| \| \| \| 4(36.4) \| \| \| \| \| \| \| \| \| \| \| \| \| \| 4(44.5) \| \| \| \| \| \| \| \| Absent \| 0(0.0) \| \| \| \| \| \| \| \| \| \| \| \| \| \| \| \| \| \| \| \| 0(0.0) \| \| \| \| \| \| 0(0.0) \| \| \| \| \| \| \| \| \| \| \| \| \| \| 0(0.0) \| \| \| \| \| \| \| \| p-value \| 0.675 \| \| \| \| \| \| \| \| \| \| \| \| \| \| \| \| \| \| \| \| \| \| \| \| \| \| 0.522 \| \| \| \| \| \| \| \| \| \| \| \| \| \| \| \| \| \| \| \| \| \| **Labial resorption** \| \| \|  \| \| \| \| \| \| \| \| \| \| \| \| \| \| \| \| \| \| \| \| \| \| \| \|  \| \| \| \| \| \| \| \| \| \| \| \| \| \| \| \| \| \| \| \| \| \| Light \| \| \| \| 2(18.2) \| \| \| \| \| \| \| \| \| \| 1(11.1) \| \| \| \| \| \| \| \| \| \| 2(18.2) \| \| \| \| \| \| \| \| \| \| \| \| \| \| 0(0.0) \| \| \| \| \| \| \| \| \| \| \| Moderate \| \| \| \| \| 1(9.1) \| \| \| \| \| \| \| \| \| \| 3(33.3) \| \| \| \| \| \| \| \| \| \| \| \| 2(18.2) \| \| \| \| \| \| 3(33.3) \| \| \| \| \| \| \| \| \| \| \| \| \| \| \| \| Severe \| \| \| \| \| 8(72.7) \| \| \| \| \| \| \| \| \| \| 5(55.6) \| \| \| \| \| \| \| \| \| \| \| \| 7(63.6) \| \| \| \| \| \| 5(55.6) \| \| \| \| \| \| \| \| \| \| \| \| \| \| \| \| Absent \| \| \| \| \| 0 (0.0) \| \| \| \| \| \| \| \| \| \| 0(0.0) \| \| \| \| \| \| \| \| \| \| \| \| 0(0.0) \| \| \| \| \| \| 1(11.1) \| \| \| \| \| \| \| \| \| \| \| \| \| \| \| \| **p-value** \| \| \| 0.398 \| \| \| \| \| \| \| \| \| \| \| \| \| \| \| \| \| \| \| \| \| \| \| \| 0.338 \| \| \| \| \| \| \| \| \| \| \| \| \| \| \| \| \| \| \| \| \| \|  \| \| \| \| \| \| \| \| \| \|  \| \| \| \| \| \| \| \| \| \| \| \| \| \| \| \| \|  \| \| \| \| \| \| \| \| \| \| \| \| \| \| \| \| \| \| \| \| \| \| **Vaginal introitus narrowing** \| \| \| \| \| \| \| \| \| \|  \| \| \| \| \| \| \| \| \| \| \| \| \| \| \| \| \|  \| \| \| \| \| \| \| \| \| \| \| \| \| \| \| \| \| \| \| \| \| \| Present \| \| \| \| \| \| 10(90.9) \| \| \| \| \| \| \| \| \| 7(77.8) \| \| \| \| \| \| \| \| \| \| \| \| 10(90.9) \| \| \| \| \| 7(77.8) \| \| \| \| \| \| \| \| \| \| \| \| \| \| \| \| \| Absent \| \| \| \| \| \| 1(9.1) \| \| \| \| \| \| \| \| \| 2(22.2) \| \| \| \| \| \| \| \| \| \| \| \| 1(9.1) \| \| \| \| \| 2(22.2) \| \| \| \| \| \| \| \| \| \| \| \| \| \| \| \| \| **p-value** \| \| \| \| \| \| 0.566 \| \| \| \| \| \| \| \| \| \| \| \| \| \| \| \| \| \| \| \| \| 0.566 \| \| \| \| \| \| \| \| \| \| \| \| \| \| \| \| \| \| \| \| \| \|  \| \| \| \| \| \|  \| \| \| \| \| \| \| \| \| \| \| \| \| \| \| \| \| \| \| \| \|  \| \| \| \| \| \| \| \| \| \| \| \| \| \| \| \| \| \| \| \| \| \| **Urethral occlusion** \| \| \| \| \| \|  \| \| \| \| \| \| \| \| \| \| \| \| \| \| \| \| \| \| \| \| \|  \| \| \| \| \| \| \| \| \| \| \| \| \| \| \| \| \| \| \| \| \| \| Present \| \| \| \| \| \| 0 (0.0) \| \| \| \| \| \| \| \| \| \| 0 (0.0) \| \| \| \| \| \| \| \| \| \| \| 0 (0.0) \| \| \| \| \| \| \| 0 (0.0) \| \| \| \| \| \| \| \| \| \| \| \| \| \| \| Absent \| \| \| \| \| \| 11(100.0) \| \| \| \| \| \| \| \| \| \| 9(100.0) \| \| \| \| \| \| \| \| \| \| \| 11(100.0) \| \| \| \| \| \| \| 9(100.0) \| \| \| \| \| \| \| \| \| \| \| \| \| \| \| **p-value** \| \| \| \| \| \| - \| \| \| \| \| \| \| \| \| \| \| \| \| \| \| \| \| \| \| \| \| - \| \| \| \| \| \| \| \| \| \| \| \| \| \| \| \| \| \| \| \| \| \|  \| \| \| \| \| \|  \| \| \| \| \| \| \| \| \| \| \| \| \| \| \| \| \| \| \| \| \|  \| \| \| \| \| \| \| \| \| \| \| \| \| \| \| \| \| \| \| \| \| \| **Perianal compromising** \| \| \| \| \| \| \| \|  \| \| \| \| \| \| \| \| \| \| \| \| \| \| \| \| \| \| \|  \| \| \| \| \| \| \| \| \| \| \| \| \| \| \| \| \| \| \| \| \| | | | | | | | | | | | | | | | | | | | | | | | | | |
| Light | | | | 0(0.0) | | | 0(0.0) | | | | | 2 (18.2) | | | | | | | 0 (0.0) | | | | | | |
| Moderate | | | | 2 (18.1) | | | 1 (11.1) | | | | | 2 (18.2) | | | | | | | 1 (11.1) | | | | | | |
| Severe | | | | 4 (36.4) | | | 1 (11.1) | | | | | 2 (18.2) | | | | | | | 1 (11.1) | | | | | | |
| Absent | | | | 5(45.5) | | | 7(77.8) | | | | | 5(45.4) | | | | | | | 7(77.8) | | | | | | |
| **p-value** | | | | | 0.318 | | | | | | | | | | 0.419 | | | | | | | | | | |
|  | | | | |  | | | |  | | | | | |  | | | | | | | |  | | |
| **Synechia** | | | | |  | | | |  | | | | | |  | | | | | | | |  | | |
| Light | | | | 8 (72.7) | | | 3 (33.3) | | | | | 8 (72.7) | | | | | | | | 3 (33.3) | | | | | |
| Moderate | | | | 0 (0.0) | | | 2 (22.2) | | | | | 0 (0.0) | | | | | | | | 1 (11.1) | | | | | |
| Severe | | | | 0 (0.0) | | | 0 (0.0) | | | | | 0 (0.0) | | | | | | | | 0 (0.0) | | | | | |
| Absent | | | | 3 (27.3) | | | 4 (44.5) | | | | | 3 (27.3) | | | | | | | | 5 (55.7) | | | | | |
| **p-value** | | | | 0.119 | | | | | | | | 0.165 | | | | | | | | | | | | | |

Source: The authors (2020).
