# Supplementary material for: Randomized clinical trial with fractional CO2 laser and Clobetasol in the treatment of Vulvar Lichen Sclerosus: a clinic study of feasibility
Source: BMC Res Notes. 2023 Mar 10;16:33. doi: 10.1186/s13104-023-06300-7 (PMC9999649; doi:10.1186/s13104-023-06300-7)
Supplement: Supplementary file 4 — Additional file 4: Table S2. Relationship between vulvar involvement before treatment and the score obtained in the Environment domain of the WHOQOL-BREF 3 months after treatment. [file 13104_2023_6300_MOESM4_ESM.docx]

**Table S2 – Relationship between vulvar involvement before treatment and the score obtained in the Environment domain of the WHOQOL-BREF 3 months after treatment**

| **Variable** | **Mean Rank (Environment Domain)** | | | | | | | |
| --- | --- | --- | --- | --- | --- | --- | --- | --- |
| ***Extent of disease*** |  |  |  |  |  |  |  |  |
| In eight  Lips + clitoris  Lips + clitoris + perineum |  |  | 12,69  12,07  4,80 |  |  |  |  |  |
| **p-value** |  |  | **0,042*** |  |  |  |  |  |
|  |  |  |  |  |  |  |  |  |

*Kruskal-Wallis test
