# Supplementary material for: Randomized clinical trial with fractional CO2 laser and Clobetasol in the treatment of Vulvar Lichen Sclerosus: a clinic study of feasibility
Source: BMC Res Notes. 2023 Mar 10;16:33. doi: 10.1186/s13104-023-06300-7 (PMC9999649; doi:10.1186/s13104-023-06300-7)
Supplement: Supplementary file 5 — Additional file 5: Table S3. Relationship between the presence of clinical signs of Lichen Sclerosus Vulvar and the patient's self-perception about the treatment. [file 13104_2023_6300_MOESM5_ESM.docx]

**Table S3 – Relationship between the presence of clinical signs of Lichen Sclerosus Vulvar and the patient's self-perception about the treatment**

| **Variable** | **Self-perception about the treatment**  **(Mean Rank)** | | | | | | | |
| --- | --- | --- | --- | --- | --- | --- | --- | --- |
| ***Clinical signs*** |  |  | **Aspect of self-perception** |  |  |  |  |  |
| **Ulceration**  Mild  Absent |  |  | **Vulva appearance**  15,50  8,97 |  |  |  |  |  |
| **p-value** |  |  | **0,052*** |  |  |  |  |  |
| **Hyperkeratosis**  Mild  Moderate  Severe  **p-value**  **Perianal involvement**  Severe  Absent  **p-value**  **Synechiae**  Mild  Absent  **p-value**  **Narrowing of the vaginal introitus**  Yes  No  p-value |  |  | **Vulva appearance**  12,95  7,79  3,00  **0,021***  **Sexual activity**  2,00  5,50  **0,031***  **Sexual activity**  5,83  2,63  **0,048***  **Pruritus**  9,44  16,50  **0,044*** |  |  |  |  |  |

*Kruskal-Wallis test
